# The complete chloroplast genome sequence of *Trapa bicornis* Osbeck (Lythraceae)

**DOI:** 10.1080/23802359.2020.1788432

**Published:** 2020-07-11

**Authors:** Fangfang Sun, Yulai Yin, Bowen Xue, Ronghua Zhou, Jun Xu

**Affiliations:** Suzhou Academy of Agricultural Science, Suzhou, China

**Keywords:** *Trapa bicornis*, chloroplast genome, Lythraceae, phylogeny

## Abstract

*Trapa* (Lythraceae) is an aquatic plant genus widely distributed in the old world. Although *Trapa* species have great edible and medical value, studies related to species identification and utilization are still lacking. Here, we reported the complete chloroplast genome sequence of a cultivated species, *T. bicornis.* The chloroplast genome size of *T. bicornis* was 155,539 bp, consisting of a pair of inverted repeat (IR) regions (24,386 bp), separated by a large single copy (LSC) region (88,493 bp) and a small single copy (SSC) region (18,274 bp). A total of 130 genes were annotated, including 85 protein-coding genes, 37 tRNA genes, and 8 rRNA genes. The phylogenomic analysis supported the monophyly of *Trapa*, and a sister relationship between *T. bicornis* and *T. natans*.

The genus *Trapa* L. (Myrtales: Lythraceae) comprises approximately 30 species, that are widely distributed in temperate to subtropical regions of Europe, Asia, and Africa (Chen et al. 2007). As its fruits are edible and rich in protein and starch, species of this genus have been widely cultivated since the Neolithic (Hoque et al. 2009; Artyukhin et al. 2019). Today, however, some taxa, e.g. *T. muzzanensis* and *T. verbanensis* have become rare and Endangered due to climatic fluctuations, changes in the drainage of many wetlands, ponds and lakes, etc. (Karg 2006; Frey et al. 2017). Besides, the taxonomy of the genus is extremely confusing worldwide because of the wide variability of morphological traits (Kim et al. 2010; Li et al. 2017). Thus, more effective molecular markers are needed to foster efforts regarding the identification, conservation, and utilization of *Trapa* species. Here, we reported the chloroplast genome sequence of *T. bicornis* Osbeck, which is the first one in cultivated *Trapa* species, and reconstructed the phylogenetic relationship with other Lythraceae species. Samples of *T. bicornis* was collected from the vegetable research institute of Suzhou, Jiangsu, China (120.5632°E; 31.3684°N). The voucher specimen was deposited in the Herbarium of Zhejiang University (HZU100718). Genomic DNA was extracted from silica-dried leaf tissue using DNA Plantzol Reagent (Invitrogen, Shanghai, China). DNA library preparation and 125-bp paired-end sequencing were performed on the Illumina HiSeq^2500^ platform. The chloroplast genome was assembled using NOVOPlasty v.2.63 (Dierckxsens et al. 2017), with *T. maximowiczii* (NC_037023) (Xue et al. 2017) as the reference. The resultant genome was annotated in Geneious R11 (http://www.geneious.com) by comparing to *T. maximowiczii*. The new annotated chloroplast genome sequence was deposited in GenBank (Accession No. MT374084).

The chloroplast genome sequence of *T. bicornis* was 155,539 bp in length and exhibited the typical quadripartite structure, consisting of a pair of IR regions of 24,386 bp, separated by a LSC region of 88,493 bp and a SSC region of 18,274 bp. The GC contents of the LSC, SSC, and IR regions are 34.2, 30.2, and 42.8%, respectively, with the overall content of 36.4%. The chloroplast genome encoded a total of 130 genes (85 protein-coding, 37 tRNA, and 8 rRNA), of which 18 (7 protein-coding genes, 4 rRNA genes and 7 tRNA genes) were duplicated. Intron-exon structure analysis indicated that 3 protein-coding genes (*clp*P, *ycf*3, and *rps*12) had two introns.

## Phylogenetic analyses

**Figure 1.**
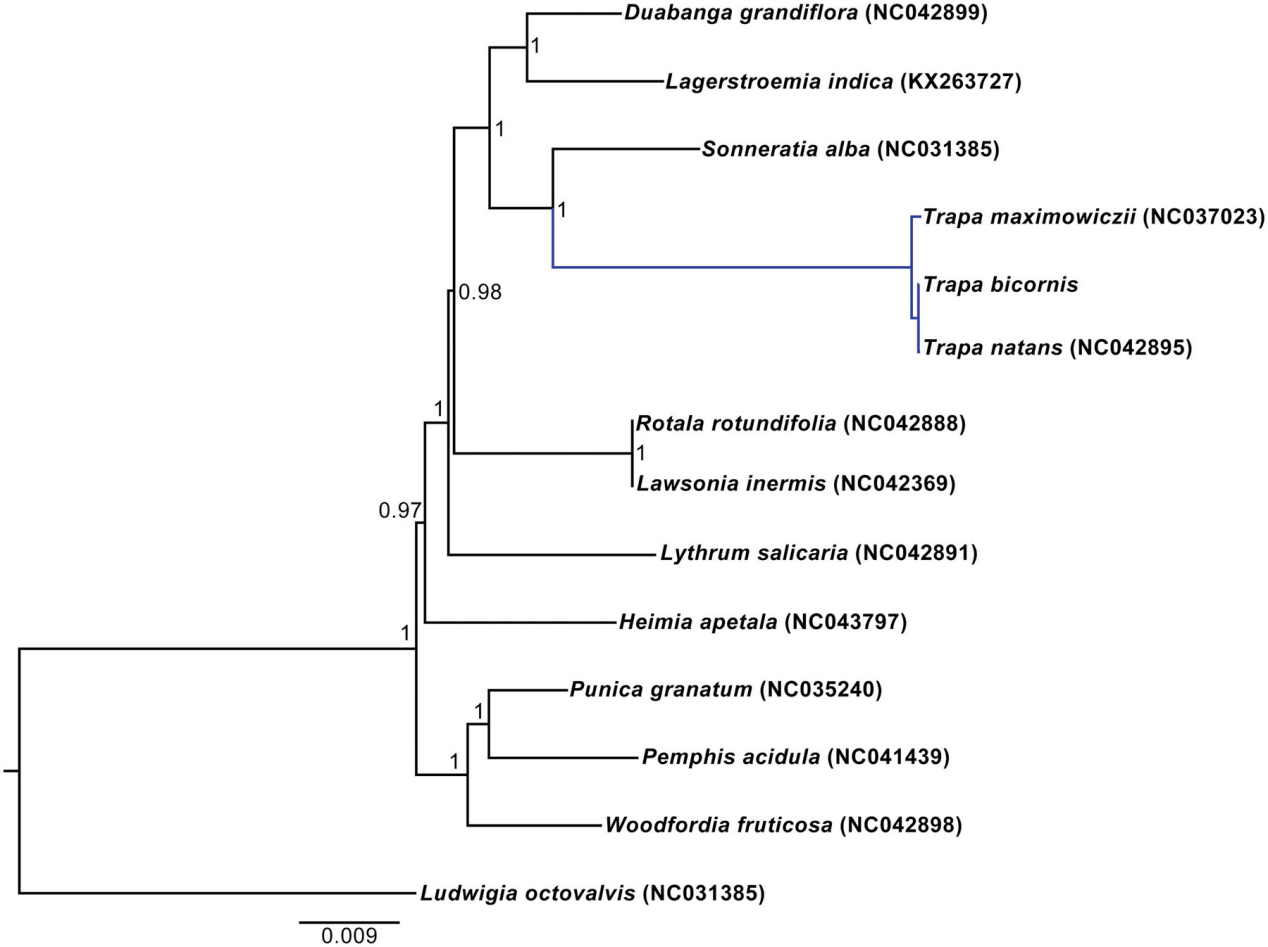
Phylogenetic tree using maximum-likelihood (ML) based on plastomes of 13 Liliaceae species with *Ludwigia octovalvis* as an outgroup. Numbers near the nodes represent ML bootstrap values.

The phylogenetic relationship of Lythraceae was reconstructed using maximum likelihood (ML) method based on the multiple alignment of reported 13 chloroplast genomes within this family, with *Ludwigia octovalvis* (NC031385) as an outgroup. ML analysis was conducted using PhyML v.3.0 (Guindon et al. 2010). The phylogenetic tree strongly supported the sister relationship of *Trapa* and *Sonneratia*, which is consistent with previous studies (Berger et al. 2016; Yu et al. 2018). Within *Trapa*, *T. bicornis* was identified as sister to *T. natans*, and these two species in turn formed a clade sister to *T. maximowiczii* (Figure 1).

## Data Availability

The data that support the findings of this study are openly available in Genbank with the accession codes MT374084 (https://www.ncbi.nlm.nih.gov/nuccore/MT374084), MT374084.
